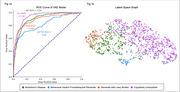# Variational autoencoder latent space as a robust and pragmatic clinical classification tool for dementia

**DOI:** 10.1002/alz.093646

**Published:** 2025-01-09

**Authors:** William C Wakefield, Leland R Barnard, Hugo Botha, Jonathan Graff‐Radford, Kejal Kantarci, Brad F. Boeve, Ronald C. Petersen, Clifford R. Jack, Val J. Lowe, David T. Jones

**Affiliations:** ^1^ Department of Neurology, Mayo Clinic, Rochester, MN USA; ^2^ Mayo Clinic, Rochester, MN USA; ^3^ Department of Radiology, Mayo Clinic, Rochester, MN USA

## Abstract

**Background:**

Many proposed clinical decision support systems (CDSS) require multiple disparate data elements as input, which makes implementation difficult, and furthermore have a black‐box nature leading to low interpretability. Fluorodeoxyglucose Positron Emission Tomography (FDG‐PET) is an established modality for the diagnosis of dementia, and a CDSS that uses only an FDG‐PET image to produce a reliable and understandable result would ease both of these challenges to clinical application.

**Method:**

A deep variational autoencoder (VAE) was used to extract a latent representation of each image through prior training from FDG‐PET brain images (n=2000). This unsupervised VAE has a novel graph convolutional architecture that makes it applicable to masked template space images. A logistic regression model was used to classify each image. A parametric study of the latent space revealed the imaging features that were used in the logistic regression model to differentiate each class. A separate dataset of participants labeled with their clinical diagnosis (n=1239) was used to assess the logistic regression model’s diagnostic accuracy of cognitively unimpaired (CU) (n=679) and between common dementia subtypes: Alzheimer’s disease (AD) (n=310), Lewy body dementia (DLB) (n=151), and behavioral variant frontotemporal dementia (bvFTD) (n=99). The logistic regression classifier was evaluated using receiver‐operator characteristic (ROC‐AUC) curves.

**Result:**

ROC‐AUC curves for CU and each dementia subtype are illustrated in Figure 1a. A k nearest neighbors model was used to develop a graphical representation of the latent space where nodes are images and edges are drawn between nearest neighbors, illustrated in Figure 1b. The graph of the latent space demonstrates a comparison between dementia subtypes and CU images.

**Conclusion:**

In this study, we developed a proof‐of‐concept VAE that can achieve differential diagnosis with 84.8% balanced accuracy. The model architecture provides visual interpretability, and shows that the hippocampi, parietal lobes, and cerebellum, were useful for distinguishing AD, the frontal lobe, posterior cingulate cortex, and hippocampi were useful for distinguishing DLB, and prefrontal pole, subcortical structures, and occipital lobe were useful for distinguishing bvFTD. These regions of metabolism align with clinical pathology and metabolic patterns.